# Bone augmentation with autologous tooth shell in the esthetic zone for dental implant restoration: a pilot study

**DOI:** 10.1186/s40729-021-00389-w

**Published:** 2021-11-08

**Authors:** Shuyi Li, Ming Gao, Miao Zhou, Yibo Zhu

**Affiliations:** 1 Department of Stomatology, Guangdong Provincial People’s Hospital, Guangdong Academy of Medical Sciences, Guangzhou, 510000 China; 2 Department of Oral and Maxillofacial Surgery/Pathology, Amsterdam UMC and Academic Center for Dentistry Amsterdam (ACTA), Amsterdam Movement Science, Vrije Universiteit Amsterdam, Amsterdam, 1081 LA The Netherlands; 3 Fourth Division, Peking University School and Hospital of Stomatology, Beijing, 100081 China

**Keywords:** Autologous tooth shell, Esthetic zone, Dental implant, Bone deficiency, Clinical study

## Abstract

**Objectives:**

To investigate the outcome and short-term follow-up of autogenous tooth shell (TS) grafting for bone augmentation in the esthetic zone, as well as stability and esthetics of implant-supported restoration.

**Materials and methods:**

A total of 8 patients with 11 implants in 11 sites were enrolled in this study. All the horizontal and/or vertical bone defects in the esthetic zone were augmented by tooth shells, which were fixed laterally to the residual bone with osteosynthesis screws. The gap between the shell and residual bone was filled with Bio-Oss® granules. Four months after bone augmentation, dimensionally sufficient dental implants were inserted and implants-supported prostheses were made 3 months later. The esthetic outcome was evaluated by pink esthetic score (PES) and white esthetic score (WES) one year after prosthetic restoration. Horizontal ridge width (HRW) was assessed before and immediately after bone augmentation, as well as 4 and 19 months post-augmentation by radiography. The stability and absorption of TS grafts were evaluated at the 4th and 19th months post-augmentation.

**Results:**

Though wound dehiscences occurred in 3 cases, secondary healings were obtained after TS modification and irrigation. The other 5 cases went through uneventful healing during the whole observation period. Radiographic examination showed that HRW was 8.01 ± 0.93 mm (median: 7.80, 95% CI 7.38, 8.64) 4 months after TS augmentation, which was statistically different compared to HRW (2.72 ± 1.73 mm) at the baseline. Mean HRW gain was 5.29 ± 2.03 mm (median: 4.60, 95% CI 3.92, 6.66). Three-dimensional bone volume in all the augmented sites was sufficient for dental implants insertion and prosthetic restoration. Follow-up of one year showed stable marginal bone around dental implants. The implant survival rate was 100%. HRW losses were 0.65 ± 0.43 mm (the 4th month) and 1.05 ± 0.54 mm (the 19th month) compared to HRW immediately after augmentation. The PES and WES of final prosthetic restorations were 8.09 ± 0.70 and 8.91 ± 0.54, respectively.

**Conclusions:**

Autogenous tooth shell grafting is a reliable approach for bone augmentation in the esthetic zone for dental implant treatment, allowing for favorable stability and esthetic outcome of implant-supported prosthesis within the one-year follow-up period.

## Introduction

Various alveolar bone defects are being witnessed during clinical treatments. Rapid reduction of 32% in horizontal bone dimension is detected in the first 3 months after tooth extraction [1]. Significant vertical bone loss (~ 7.5 mm) occurs within 2 months when the thickness of buccal bone is less than 1 mm (thin-wall type) [2]. Horizontal, vertical, or combined defects significantly influence dental implant placement and its long-term stability, as well as the final esthetics outcome. Due to its osteogenic property, autogenous bone (AB) is considered as the gold standard of graft materials in alveolar bone defect repair [3]. However, drawbacks associated with AB are unavoidable, e.g., limited supply, trauma in donor site and undesired absorption [4, 5]. As a result, alternative bone grafts derived from different origins are being used. They can reduce operative time with low morbidity and have extensive sources [6]. However, to maximally ensure safety and reduce risks of disease transmission and immune rejection, xenogeneic/allogenic bone grafts need to be processed which may devitalize their bioactivities. Synthetic bone substitutes are also not satisfactory with insufficient bone formation ability [7]. Therefore, continuous efforts have been taken to develop a more appropriate bone graft material for the clinic.

Recently, autogenous tooth shell (TS) from dentin has attracted much attention due to lower cost and less invasiveness than AB, as well as its components similarity to bone tissue [8]. Besides, TS has osteogenic and osteoconductive properties as AB. It shows that dentin possesses osteogenesis-related growth factors extensively distributed in extracellular matrix (ECM) [9]. These osteogenic factors include bone morphogenetic proteins (BMPs) and transforming growth factor-β (TGF-β), which can induce mesenchymal stem cells to differentiate into osteoblasts and stimulate proliferation and differentiation of osteoblasts, thereby promoting bone formation [10]. Dentin ECM consists of 89% type I collagen, which is also the main component of bone tissue. It can serve as a template for bio-mineralization and bone formation. Meanwhile, bone sialoprotein (BSP) in ECM participates in bio-mineralization process during ossification by binding to collagen and regulating nucleation of hydroxyapatite (HA) [11]. And OPN in dentin ECM concentrates in mineralized collagen matrix and increases cells’ adhesion to HA [12]. Furthermore, the mineralized components (~ 70%) of TS will release calcium and phosphate during degradation, which are essential elements for bone regeneration. Therefore, dentin is a promising alternative to AB. When employing TS and AB for alveolar bone augmentation in dogs, median osteocalcin expression of TS is 2.5 times higher than AB. Meanwhile, more regenerated bone volume is observed in TS than that of AB [13]. A series of clinical studies show satisfying results in alveolar bone augmentation, which make it possible to place dental implants [14, 15]. Particulate dentin (AutoBT, Korea Tooth Bank Co., Seoul, Korea) can be used in maxillary sinus lifting, which shows comparable bone regeneration outcome with Bio-Oss® except that more trabecular thickness and osteoid are observed in AutoBT [16]. However, all these studies did not pay specific attention to alveolar bone augmentation in the esthetic zone, and lacked the outcome and follow-ups of augmented bone and implants after load.

Compared with the posterior zone, rehabilitation of maxillary teeth and bone is much challenging and complicated in the esthetic zone [17]. The three dimensions of bone contour, quantity and quality will directly influence dental implant position, stability, soft tissue volume and final restoration outcome. When a maxillary anterior tooth is missing, significant loss of buccal bone happens within a few weeks [18], which results in depressive bone contour, shortage of keratinized gingiva and brings esthetic challenges. Thus, a study of TS on the clinical effectiveness of bone augmentation in the esthetic zone is of paramount importance.

The aim of this pilot study was to investigate the clinical outcome and follow-up of TS for alveolar reconstruction in the esthetic zone, which will give more scientific proof for TS to be an alternative to traditional autologous bone graft in future.

## Material and methods

### Study design

In this proof-of-concept study, a total of 8 patients (age: 24–48) were enrolled. They all had severe bone defects in edentulous sites and planned to receive implant-supported prostheses. The bone defects exhibited horizontal and/or vertical dimension losses. This clinical study was approved by the ethics committee of Peking University (No. PKUSSIRB-201841200), China. Before surgery, every patient was informed of the treatment in details and signed informed consent.

### Inclusion criteria

- Age between 20 to 60 years;
- Insufficient bone contour for dental implant placement simultaneously;
- Volunteer for participating alveolar bone augmentation by tooth shell technique;
- With non-retainable wisdom tooth;
- Healthy oral mucosa;

### Exclusion criteria

- Age < 20 or > 60 years;
- Smokers;
- Severe or progressive periodontal diseases;
- Contraindications for bone augmentation and dental implant treatment;

### Outcome evaluations

One therapeutic endpoint was to obtain sufficient bone width and height for dental implants without the need for a secondary intervention. Initial ridge width was set as baseline (HRW 0). HRW after bone augmentation surgery (HRW 1) and re-entry at the 4th month (HRW 2) was measured to calculate bone width gain (HRW 2–HRW 0) and HRW loss (HRW 1–HRW 2). Reference site was set as the point where two lines met: one was in the center of edentulous zone, parallel to the adjacent tooth; the other was parallel to the occlusal plane, 3 mm below cemento-enamel junctions of adjacent single teeth on both sides of edentulous zone. HRW 0, 1, 2 were measured by a standard caliper. 

The other endpoint was to assess short-term stability of dental implants, bone volume, pink esthetic score (PES) and white esthetic score (WES) 1 year after prosthesis. Cone-beam computed tomography (CBCT) was used to assess bone contour of the alveolar ridge before and after each surgical treatment, as well as osseointegration in the follow-up study. Stability of augmented bone after 19 months (HRW 3) was calculated as (HRW 1–HRW 3). HRW 3 was measured on a sagittal plane parallel to the long axis of adjacent teeth by CBCT. Status of wound healing/soft tissue dehiscences/infection was also recorded. 

### Treatment procedures

**Fig. 1:**
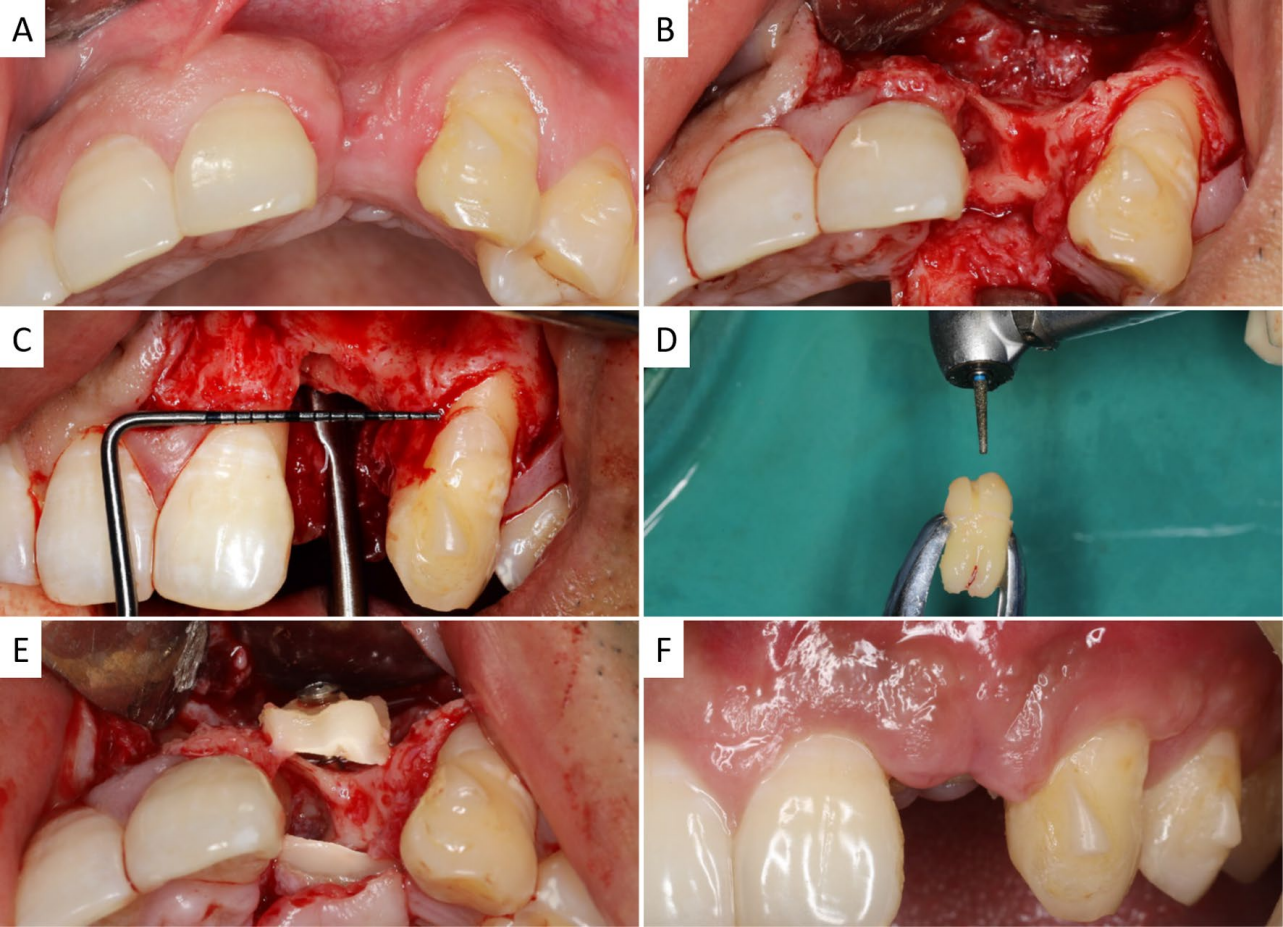
Illustration of bone augmentation surgery using tooth shell (TS) technique in the esthetic zone. **A** Occlusal view before the operation; **B**–**C** exposure of deficient ridge, occlusal view (**B**) and buccal view (**C**) of the defect; **D** modification of extracted wisdom tooth; **E** rigid fixation of TS in defect site; **F** uneventful healing of soft tissue

Prominently depressed labial bone was observed on a gross view (Fig. 1A). Under sterilized conditions, the patient received local anesthesia. With a mid-crestal incision in the edentulous site and a vertical incision at the distal axial angle of the adjacent tooth, full-thickness mucoperiosteal flaps were elevated and the alveolar ridge was exposed (Fig. 1B). After removal of the granulation tissues, severe horizontal (10 mm in width indicated by a periodontal probe) and vertical bone losses were observed (Fig. 1C). The wisdom tooth was gently extracted and thoroughly rinsed. Detectable debris of the tooth was eliminated (Fig. 1D) followed by the removal of the enamel and cementum part of the tooth by a coarse diamond bur under sterile saline (0.9%) cooling. Then, the tooth was separated longitudinally by a diamond disk. The pulp was also removed during this procedure. As to the defect size and desired contour of the alveolar bone, a diamond bur was used to obtain tooth shells (thickness of 1–1.5 mm), which were adapted to cover labial and/or palatal defects. Subsequently, TS grafts were predrilled and rigidly fixed to residual host bone using one or two osteosynthesis screws (1.5 × 10 mm, Xi’an Zhongbang Co., Ltd, China) (Fig. 1E). Bio-Oss® (Geistlich Pharma AG, Wolhusen, Switzerland) was used to fill the gaps between TS and host bone. The bone grafting site was then covered by an absorbable collagen membrane (Bio-Gide®, Geistlich Pharma AG, Wolhusen, Switzerland). Finally, the periosteum of the buccal flap was cut and separated to get a tension-free wound closure. Patients were provided with antibiotics after the surgery for 7 days and instructed for oral health care to get primary soft tissue healing (Fig. 1F).

**Fig. 2:**
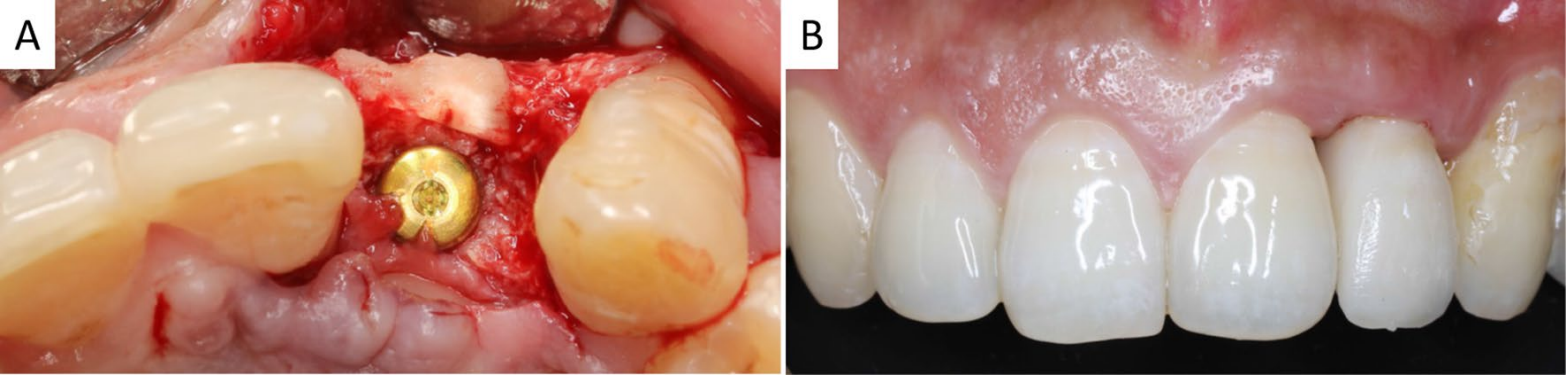
Re-entry surgery of dental implant placement and prosthesis. **A** Submerged healing of dental implant in the augmented alveolar bone. Labial TS still existed and had integrated with host bone; **B** final crown restoration

Four months later, partial-thickness flaps were elevated and the osteosynthesis screws were removed. The implant site was prepared and a commercially available dental implant (SPI® ELEMENT INICELL, Thommen Medical AG, Grenchen, Switzerland) was inserted in a prosthetically driven position with submerged healing (Fig. 2A). Three months later, a conventional re-entry surgery was performed and then the implant-supported prosthesis was delivered (Fig. 2B).

### Esthetic outcome evaluation

All the implant-supported crowns were photographed and the contralateral teeth were also presented. For the incisors, symmetric images were taken referring to the midline. PES and WES analyses were done by an experienced prosthodontist. Each implant site was scored ranging from 0 to 10 [19].

### Statistical analysis

Data were shown as mean ± standard deviations. Statistical analysis was done by IBM SPSS Statistics software 20. Normal distribution was examined before using paired-samples t-test to analyze bone width changes and bone graft resorption. The significant difference was set as *p* < 0.05.

## Results

**Fig. 3:**
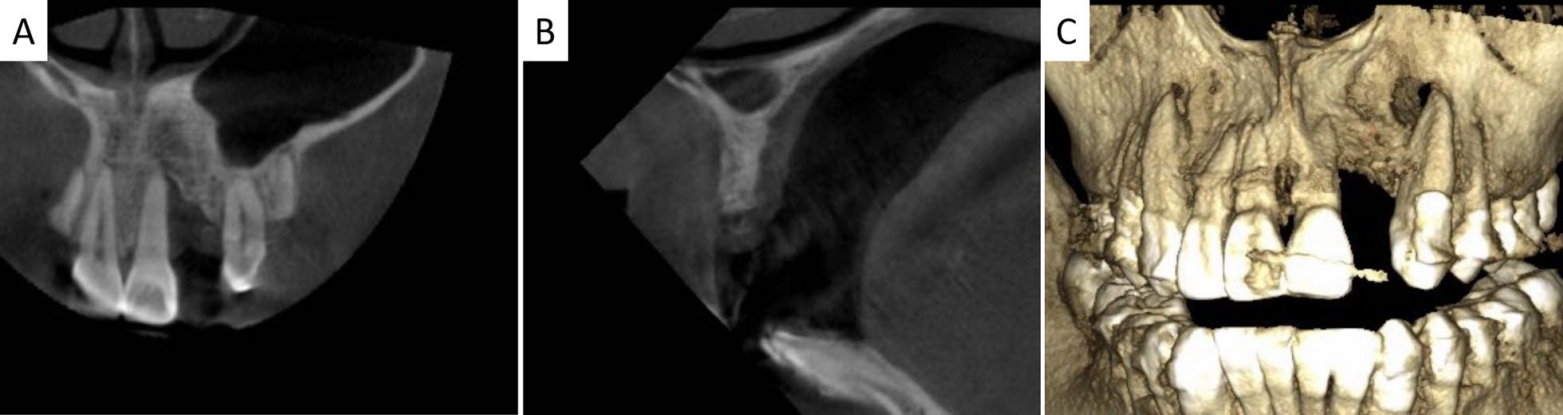
CBCT examination before surgery. **A**–**C** showed coronal-, sagittal plane and 3D reconstruction views

Before bone augmentation surgery, CBCT showed a severe alveolar bone loss from coronal- and sagittal plane and 3D reconstruction views in edentulous site (Fig. 3). Five cases went through uneventful healing while gingival dehiscences occurred in the other 3 cases. Considering that the wounds showed no sign of infection, under local anesthesia, the exposed TS grafts were modified by a diamond bur. The wounds were rinsed alternately with sterile saline (0.9%) and hydrogen peroxide (3%). With a full reduction of soft tissue tension, the wounds closed naturally, which got secondary wound healing.

**Fig. 4:**
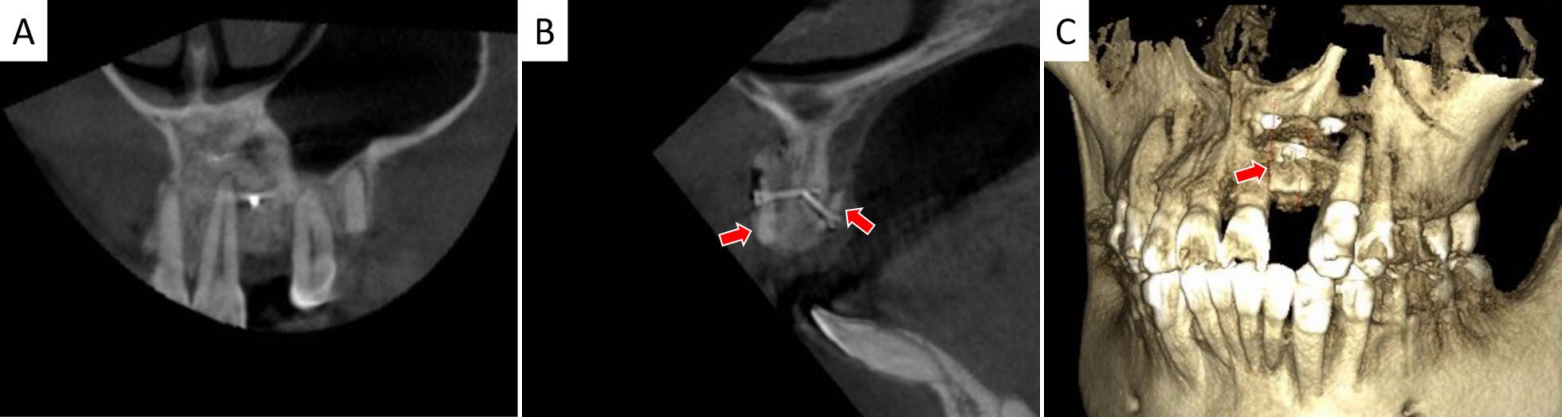
CBCT examination 4 months post-operation. **A**–**C** showed coronal-, sagittal plane and 3D reconstruction views. Red arrows depict TS integrated with host bone

**Fig. 5:**
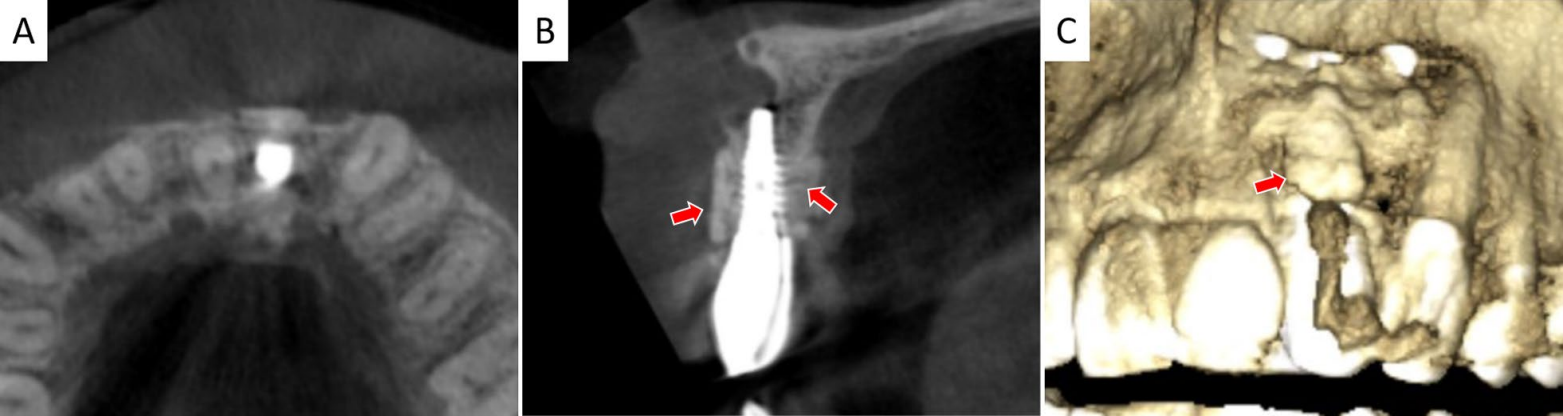
CBCT examination 1 year after implant-supported prosthesis. **A**–**C** showed coronal-, sagittal plane and 3D reconstruction views. Red arrows depict TS still existed

Four months post-operation, TS of high density still existed and had integrated with host bone (Fig. 4). Details of the edentulous zone, HRW gain and TS resorption are listed in Table 1. The final augmented bone width (HWR 2) was 8.01 ± 0.93 mm, which was statistically different from baseline (HRW 0, 2.72 ± 1.73 mm) (*p* < 0.0001). The final mean HRW gain was 5.29 ± 2.03 mm. Compared to HRW after surgery (HRW 1), slight TS resorption in horizontal was detected (0.65 ± 0.43 mm) (*p* < 0.001). Thus, dimensionally sufficient standard dental implants were inserted. Upon 1-year follow-up, no peri-implant radiolucency was detected and thick alveolar bone surrounded the dental implants. TS still existed and integrated with recipient’s bone. The grafted alveolar bone still underwent remodeling that a decrease of HRW (1.05 ± 0.54) mm was measured (*p* < 0.0001 compared with HRW 1). Implant–bone integration and the marginal bone level were well preserved (Fig. 5). Healthy peri-implant hard and soft tissues were also evidenced. Esthetic evaluations of PES and WES were 8.09 ± 0.70 and 8.91 ± 0.54, respectively.

**Table 1:** Clinical records of staged treatment, implant size, HRW changes, GR resorption (in mm) and esthetic evaluations

Patient no.	Tooth position	HRW 0	HRW1	HRW2	HRW3	Implant type	HRW gain	GR1	GR2	PES	WES
1	11	4.4	8.5	8.5	7.2	4.0 × 11	4.1	0.0	1.3	8	10
2	13	4.3	10.0	9.3	9.1	4.5 × 9.5	5.0	0.7	0.9	9	9
3	11	1.5	7.1	6.9	6.8	4.0 × 9.5	5.4	0.2	0.3	8	9
	21	2.8	7.3	7.1	6.9	4.0 × 9.5	4.3	0.2	0.4	8	9
4	11	1.7	8.2	7.5	7.5	4.0 × 11	5.8	0.7	0.7	7	8
	12	0.0	9.0	7.8	7.8	3.5 × 11	7.8	1.2	1.2	7	8
5	22	0.0	11.0	10.0	9.4	4.0 × 9.5	10.0	1.0	1.6	8	9
6	23	3.2	8.3	7.8	7.4	4.0 × 11	4.6	0.5	0.9	9	9
7	11	3.0	9.0	7.6	6.8	4.0 × 11	4.6	1.4	2.2	8	9
8	11	5.0	8.0	7.5	7.1	4.0 × 11	2.5	0.5	0.9	9	9
	21	4.0	8.8	8.1	7.7	4.0 × 11	4.1	0.7	1.1	8	9
Mean	/	2.72	8.65	8.01	7.61	/	5.29	0.65	1.05	8.09	8.91
SD	/	1.73	1.12	0.93	0.88	/	2.03	0.43	0.54	0.70	0.54
Median	/	3.00	8.50	7.80	7.40	/	4.60	0.70	0.90	8.00	9.00
95% CI	/	1.56, 3.88	7.90, 9.41	7.38, 8.64	7.02, 8.20	/	3.92, 6.66	0.36, 0.94	0.68,1.41	7.62, 8.56	8.55, 9.27

HRW, horizontal ridge width; HRW 0 and HRW 1, immediately before and after bone augmentation surgery; HRW 2 and HRW 3, the 4th and 19th month post-bone augmentation surgery; GR, graft resorption. Within-group comparisons (paired t-test): HRW gain = HRW 2–HRW 0, *p* < 0.0001; graft resorption at the 4th month (GR1) = HRW 1–HRW 2, *p* < 0.001; graft resorption at the 19th month (GR2) = HRW 1–HRW 3, *p* < 0.0001

## Discussion

In this study, the efficacy and esthetic outcome of TS grafting for horizontal alveolar ridge augmentation in the esthetic zone were evaluated. By staged treatment, severely deficient ridges were fully reconstructed, which made it possible for ideal prosthetic-driven implant placement. Follow-up of 1 year witnessed volumetric bone stability around dental implants and a favorable white and pink esthetic outcome. These findings showed TS technique is a promising method in reconstructing severe alveolar bone defects in the esthetic zone. 

Sufficient bone volume is decisive to an implant-supported prosthesis. TS has been used to augment alveolar bone defect, from an initial RW of 4.53 ± 1.54 mm to the final RW of 10.20 ± 1.70 mm at the 26th week. The average RW gain is 5.53 ± 1.88 mm, which is higher than that of AB (3.39 ± 1.41 mm) [20]. It is also efficacious in enlarging extraction socket, with RW ranging from 5.96 ± 3.99 mm before surgery to 12.25 ±  3.17 mm after augmentation. The final RW gain is 4.89 ±  2.29 mm at the 26th week [14]. Comparatively, our study got a mean RW gain of 5.29 ±  2.03 mm with an initial RW (HRW 0) of 2.72 ±  1.73 mm to 8.01 ±  0.93 mm (HRW 2) at the 4th month, which ensures the placement of a dimensionally standard dental implant. A comparative study shows that mean RW gains after autologous and Bio-Oss® block graft are about 4 mm and 5.6 mm, respectively, 6 months post-operation [21]. Our previous study used particulate coral hydroxyapatite sheltered by a titanium mesh obtained RW gain of 4.2 ±  0.5 mm in the anterior zone after 6 months [22]. These studies show a comparable RW gain to TS technique, which further demonstrate the effectiveness of using TS to restore severe bone defect.

To ensure the final RW gain and stability of implants, TS should also maintain its volumetric stability. TS is reported to have significantly less resorption than AB (0.13 ±  0.97 mm versus 1.03 ±  1.15 mm) 26 weeks post-surgery [20]. We also observed that 0.65 ±  0.43 mm of TS was absorbed after 4 months, which was less than that of the aforementioned AB. Besides, TS’ resorption after 19 months was 1.05 ±  0.54 mm, approximating to AB’s resorption at the 26th weeks. From a histological perspective, direct contact of tooth surface with bone tissue will form dento-alveolar ankylosis, which initiates ankylotic resorption with new bone tissue replacing enamel and dentin progressively [23–25]. Specifically, osteoclastic resorption islands occur on TS surface and a non-mineralized, well-vascularized tissue will replace TS first and then be replaced by woven bone [24, 26]. The density and rigidity of TS also retard the resorption process [27]. In contrast, a much quicker remodeling process happens in the transplanted autologous bone block, which will go through avascular necrosis, and then bone absorption is activated followed by creeping substitution. This process can take place at both the peripheral area of the graft and the penetrated screws area [24, 28].

TS used in this study is from the host’s wisdom tooth and it can be used as a block graft [15] or in particulate form [29]. There are three dentin derivates: mineralized dentin, partially demineralized dentin and completely demineralized dentin [30]. Mineralized dentin has excellent osteoconduction properties and certain osteoinductivity [27, 30]. Histological study shows new bone forms on both the outer and inner surfaces of dentin [27]. It can maintain bone contour and effectively restore horizontal or vertical bone defect, making it fit for cases in need of a solid osteoconductive scaffold [30]. On the contrary, demineralized dentin matrix shows low osteoconduction and high bioactivity because the removal of mineralized components leads to enlarged dentinal tubules, exposure of collagen and proteins being released [31, 32]. Clinical trials demonstrate that demineralized healthy tooth and tooth after endodontical root canal therapy can obtain comparable bone formation in alveolar socket preservation [33]. Similarly, Elfana et al. show that autogenous whole tooth graft (AWTG) and autogenous demineralized dentin graft (ADDG) are equally effective at reducing dimensional losses in alveolar ridge preservation. No significant difference is detected though ADDG promotes more new bone formation than AWTG by histological analysis [34]. The production of demineralized dentin is time-consuming, cost-intensive and complicated, which restricts its widespread use in clinics. Periodontally infected tooth root can also serve as an alternative after thorough debridement, plane, removal of cementum in contact with host bone [35, 36]. For non-retainable contaminated teeth, autoclavation (15 mins, 134 °C) is used for decontamination. In vivo studies of beagle’s vertical alveolar ridge augmentation show equally augmented bone of autoclaved tooth and natural tooth. But bone-to-implant contact of the natural tooth is much higher than autoclaved teeth [37, 38].

Unlike other studies with the re-entry of 24 to 26 weeks [14, 15, 20], re-entry at the 4th month in our study also witnessed a sufficient bone gain, and TS had integrated with recipient’s bone. Continuous observations of 1, 3 and 6 months show new bone deposited on tooth graft from the 3rd month [23]. Thus, re-entry at the 4th month is safe and time-saving for clinical treatment. Though TS augmentation allows for simultaneous dental implantation [39, 40], high risks of complications will occur, especially graft exposure. Graft exposure also happens during AB grafting and it will result in incomplete integration of graft, graft resorption and host site infection. A secondary bone augmentation will be required [41]. Allografts augmentation also encounters 18 of 137 (13%) bone grafts infection and up to 30% soft tissue complications at the graft sites, especially incision line opening and membrane exposure [42]. Though 3 cases in our study did not obtain primary healing, the exposed wounds healed well after thorough rinsing and further trimming of TS. No additional bone graft was needed during re-entry. We propose that autogenous TS can tolerate small exposure and bring predictable outcomes.

When restoring missing teeth in the anterior region, one of the key criteria is pink and white esthetics. PES represents soft tissue appearance around the prosthesis. A satisfactory PES is a result of proper surgical treatment and stable bone contour. WES mainly describes implant-supported crowns. The threshold of clinical acceptability is PES/WES > 12 and each index > 6 [19]. Buser et al. use autogenous bone chips and deproteinized bovine bone mineral to guide bone regeneration simultaneously during dental implants placements. The PES and WES of the implant-supported restoration after 1 year are 8.1 and 8.65, respectively [43, 44]. Schlee et al. get an average PES of 7.5 ± 2.6 when using bone block from the external oblique line of ramus to repair bone defect in edentulous region, which is restored by implant-supported prostheses after 4–7 months’ healing period [45]. Our study also showed a successful esthetic outcome with WES of 8.91± 0.54 and PES of 8.09 ±  0.70. The mean WES was slightly higher than the mean PES because WES mainly depends on the experience of dental technicians. The favorable PES evaluation indicates correctly placed implants and sufficient bone height and thickness, suggesting TS is a reliable bone graft. 

Though all the cases showed favorable outcomes using TS as a bone graft, there are still some limitations in our study. Long-term follow-ups are needed to track TS and bone contour changes around the implants. We will recruit more clinical cases to verify if TS can replace autologous bone in repairing different bone defect types in the esthetic zone and establish a detailed operational procedure.

## Conclusion

This pilot study demonstrated (1) TS had the capability to reconstruct severe alveolar bone defect in the esthetic zone; it showed high resistance to oral exposure; (2) short-term stability of dental implants and augmented bone contour; and (3) satisfying esthetic outcome after 1 year's follow-up, suggesting that TS grafting is a feasible approach for restoring alveolar bone defect in the esthetic zone, allowing for satisfied esthetic outcome of implant-supported prostheses.

## Data Availability

The datasets used and/or analyzed during the study are available from the corresponding authors on reasonable request.

## Abbreviations

TS: Tooth shell
PES: Pink esthetic score
WES: White esthetic score
HRW: Horizontal ridge width
AB: Autogenous bone
ECM: Extracellular matrix
BMPs: Bone morphogenetic proteins
TGF-β: Transforming growth factor-β
BSP: Bone sialoprotein
HA: Hydroxyapatite
CBCT: Cone-beam computed tomography
GR: Graft resorption
AWTG: Autogenous whole tooth graft
ADDG: Autogenous demineralized dentin graft
